# Advances in Infant and Pediatric Feeding and Nutrition

**DOI:** 10.3390/nu17142378

**Published:** 2025-07-21

**Authors:** Jann Foster

**Affiliations:** 1 School of Nursing & Midwifery, Western Sydney University, Locked Bag 1797, Penrith, NSW 2751, Australia; j.foster@westernsydney.edu.au; 2 School of Nursing and Midwifery, University of Canberra, Bruce, ACT 2617, Australia; 3 Ingham Research Institute, Liverpool, NSW 2170, Australia; 4 NSW Centre for Evidence-Based Health Care: A JBI Centre of Excellence, Parramatta, NSW 2751, Australia

This Special Issue presents original works and reviews that delve into how specific feeding strategies, spanning from lactation to complementary feeding in infancy, impact growth and neurofunctional development.

The twelve articles cover a broad spectrum of topics, ranging from the epigenetic effects of human milk on the ongoing neurodevelopment of infants and children, the impact of milk oligosaccharides, maternal fish consumption, and the use of bovine colostrum or infant formula from hydrolyzed whey protein to the validation of an instrument for evaluating parental feeding behaviors, breastfeeding, and childhood caries. These articles provide novel insights into the complex nature of infant feeding and nutrition.

Feeding infants human milk has many benefits that are universally recognized, and human milk contains all the nutrients an infant requires in the first six months of life [1]. Gialeli et al., 2023, offer fresh perspectives in their narrative review, which explores how bioactive components in breast milk, such as microRNAs, long non-coding RNAs, stem cells, and the microbiome may impact the neurodevelopment of preterm and full-term infants through epigenetic mechanisms. The review establishes a compelling connection between early-life experiences and long-lasting health outcomes in preterm and term infants.

Fan et al., 2023, reviewed the impact of milk oligosaccharides, a group of complex carbohydrates, on the brain and on neurocognitive development in early life. The authors report that the benefits for cognitive development of human milk oligosaccharides (HMOs) may be due to sialic acid and fucose, which have been implicated in brain development. Ultimately, this review posits that there is a consistent connection between early life consumption of HMOs and cognitive developmental outcomes. The review also emphasizes the importance of clinical studies to explore the specific mechanisms through which milk oligosaccharides enhance learning and memory development in infants.

Kasamatsu et al.’s 2023 cross-sectional study examined the links between maternal diet, infant feeding practices, and serum levels of docosahexaenoic acid (DHA), a crucial n-3 long-chain polyunsaturated fatty acid (LCPUFA) important for infant brain development. Data were gathered through a maternal dietary questionnaire and serum fatty acid levels from the blood samples of infants. The study found significant positive associations between the infants’ serum DHA levels and the consumption of “blue-back fish” and “white fish”. These novel results suggest that lactating mothers who regularly consume these types of fish, while also prioritizing breastfeeding over DHA-supplemented cow’s milk formula, could effectively boost their infants’ serum DHA levels. The study was conducted in Tokyo, Japan, and the authors recognize that these findings may not apply to other populations.

Whilst exclusive breastfeeding for the first 6 months of an infant’s life is recommended by the WHO, human milk continues to provide significant nutritional and immunological value beyond 6 months. The American Academy of Pediatrics supports continued breastfeeding, along with complementary foods introduced at about 6 months [2]. The literature review by Froή & Orczyk-Pawilowicz, 2024, examines the evidence supporting extended breastfeeding beyond six months. A key takeaway from the review is that longer breastfeeding durations are linked to a variety of health benefits, such as a reduced risk of gastrointestinal and respiratory infections, enhanced growth and cognitive development, and a decreased likelihood of developing allergic diseases and obesity in later life. Moreover, breastfeeding has positive effects on metabolic syndrome, blood pressure regulation, otitis media, and malaria. Based on these findings, the authors advocate for encouraging mothers to breastfeed for longer periods.

The systematic review and meta-analysis by Shrestha et al. synthesized evidence to determine if there is an association between breastfeeding and early childhood caries (ECC), a significant chronic disease that affects infants and preschool children worldwide [3]. Whilst the review did not demonstrate an overall difference in dental caries between breastfed and non-breastfed infants, a significant increase in ECC was found for children who were breastfed for ≥6 months compared to those breastfed for <6 months, those who were breastfed for ≥12 months compared to those fed for <12 months, and those who were breastfed for ≥18 months compared to <18 months. Nocturnal breastfeeding also increases the risk of ECC. This review has improved our understanding of the effect of breastfeeding exposure time on ECC. The authors recommend that policy-makers develop an infant oral health promotion program and that healthcare professionals receive training on oral hygiene practices for infants.

Whilst human milk is regarded as the best source of nutrition during early life [4], it is not always possible to rely on it exclusively, and various infant formulas and/or supplements may be used to substitute human milk. Hill & Buck’s (2023) double-blinded RCT explored the impact on serum metabolite levels of feeding formulas enriched with different levels of 2′-fucosyllactose (2′-FL) and galactooligosaccharides (GOSs), comparing them to a non-randomized group of breastfed infants. The control formula contained only GOS. The researchers hypothesized that, due to 2′-FL’s known role as a prebiotic that can influence gut microbiome composition, its supplementation could significantly affect metabolic output, mimicking some of the systemic benefits associated with breastfeeding. Their results showed that fortifying infant formula with 2′-FL led to a dose-dependent increase in circulating metabolites produced by gut microbial metabolism. Specifically, 2′-FL supplementation was linked to higher levels of secondary bile acids and the activation of systemic immune mediators, when compared to the control formula, reaching concentrations akin to those seen in breastfed infants. The researchers also offer valuable recommendations for future investigations.

Canbolat et al.’s 2024 review examines the composition, benefits, and effects of bovine colostrum (BC), and concludes that bovine colostrum is the most viable alternative to human colostrum for infant feeding. With its anti-inflammatory, antioxidant, antibacterial, prebiotic, and antiviral properties, BC offers a distinct colostrum profile. However, the authors emphasize that BC should be regarded as a supplement rather than as a reliable treatment, and its use must be guided by a healthcare professional, especially in preterm infants or those with medical conditions. The review also suggests that future research should focus on identifying the optimal dosages, formulations, and safety profiles of BC, particularly regarding allergic reactions in infants with cow’s milk protein allergies or lactose intolerance.

Protein is essential for the growth and development of infants [5]. Fleddermann et al., 2023, assess the nutritional safety and suitability of an infant formula manufactured from extensively hydrolyzed protein (HP) compared to infant formula manufactured from intact protein (IP—with a low or standard protein content). The authors conduct a combined analysis of raw data from two randomized infant feeding studies. The results show no significance in weight gain between the two formulas, and secondary growth measures such as weight, length, and head circumference were generally similar across both the HP and IP formula groups, at both protein levels. However, the HP formula group showed greater monthly gains in head circumference (for the low-protein formula, both in the per-protocol set (PPS) and the full-analysis set (FAS)) and in length (for the standard protein formula, FAS) compared to the IP formula groups. The authors note a higher occurrence of adverse events for the HP formula group, though these events were transient and mild, leading them to conclude that there were no safety concerns.

Inappropriate complementary feeding (CF) can result in childhood illness and affect a child’s growth and development [6]. The cross-sectional study by Ashraf et al. investigates the prevalence of inappropriate CF and the factors influencing early feeding patterns among mothers in Pakistan. This crucial research reveals that 47.0% of caregivers began CF before 4 months of age, which negatively impacts infant health. In contrast, 39.7% followed guidelines by introducing CF between 4 and 6 months, potentially enhancing infant health outcomes, and 30.9% started complementary feeding after 7 months. This study identifies several factors, such as birth order, mother’s employment status, parental education, the number of children, household income, maternal knowledge, and maternal health, as significant influencers of CF practices. The findings contribute to existing knowledge and highlight the need for targeted interventions and policy changes to improve CF practices in Pakistan.

Unhealthy eating behaviors in childhood contribute to the obesity epidemic [7,8]. González-Torres et al. investigated the effectiveness of the self-administered Scale on Parental Feeding Behaviors in terms of its ability to assess various parental feeding practices among Mexican caregivers, grounded in theoretical concepts of coercive control, structure, and autonomy support. The authors found that the scale is effective at measuring both the positive and negative parental eating behaviors that influence the development of healthy eating habits in childhood. Notably, the scale was deemed valuable for designing interventions aimed at preventing health issues linked to poor nutrition and childhood obesity.

Guevara et al. reviewed the nutritional composition of cereal-based foods offered to infants (from 4 months) and toddlers in Spain and Ecuador. A total of 127 products were included, with 105 from Spain and 22 from Ecuador. The study revealed that, in Ecuador, commercial companies recommend starting the consumption of cereals at 6 months, while in Spain, the recommendation is from 4 months. Only 39 products in Spain and 2 products in Ecuador could be classified as “low in sugar”. There was noticeable variation in the declaration of vitamin content between the two countries and, regarding mineral content, cereals in Ecuador had higher reported calcium levels, while Ecuadorian cereals and cookies also had higher iron content. Cookies in both countries were found to have high sodium levels. The authors highlight some differences in the nutritional composition of cereals for breastfeeding infants between those from Spain and those from Ecuador. Importantly, the authors highlight the lack of studies on the nutritional quality of infant foods in Ecuador, suggesting their research could serve as a foundation for future investigations.

This editorial summarizes the twelve articles that comprise the Special Issue further advancing our knowledge of infant and pediatric feeding and nutrition. The findings from these articles deepen our understanding of issues related to feeding and nutrition and pave the way for practical applications and future research.
